# The Growth and Scope of Voluntary Hospitals in London

**Published:** 1897-08-07

**Authors:** 


### The Institutional Workshop.

### THE GROWTH AND SCOPE OF VOLUNTARY HOSPITALS IN LONDON.

From a Correspondent.

### III.—THE EARLIER SPECIAL HOSPITALS.

The first special hospital, in the modern sense of the word, founded in London was that known as the Middlesex County Hospital for Small-pox in 1746. There was nothing novel in opening a refuge for sufferers from an epidemic. Some of the earliest hospitals of the world originated for this purpose. The new undertaking, however, had a double aim. Small-pox had long been endemic in London; small-pox patients were excluded at that time from all other hospitals, and the project was not merely a temporary expedient but designed for the permanent relief of sufferers. But it was also resolved "that the study of the practical method of treating the disease should be made a principal object, so that the institution might in time attain a national character for the most correct medical and surgical practice of the disease." It was to establish, in fact, a small-pox "school." The hospital, though liberally supported, had a chequered career. Its promoters were strong advocates for "inoculation," and the institution was used as a propaganda for the dissemination of their principles. The work of the hospital was early divided into the care of those who fell sick of the small-pox in "the natural way," and of those who voluntarily submitted to the mild form administered by inoculation. These two classes of patients were received in separate buildings, and immense opposition was, not unnaturally, raised in Islington, Clerkenwell, and Bloomsbury, where sites were procured. The dread of infection being brought into the neighbourhood was heightened by the popular clamour against inoculation as "an attempt against the prerogative of the Almighty," and more than once riots ensued. The institutions prospered, in spite of many changes of locality, and, on the introduction of vaccination at the close of the century, did good service, under Dr. Woodville, by affording opportunity for researches and experiments.

In the same year which saw the establishment of the hospitals for small-pox and inoculation, the Lock Hospital was instituted in the immediate vicinity of St. George's for a class of sufferers then excluded from most other hospitals by the terms of their constitution. The medical study of the disease, so strong an element in the establishment of the small-pox hospital, does not appear to have been an important part of the project. The aim was reformation as well as cure, and some years later the scope of the institution was enlarged by the establishment of an asylum for the reception of such patients as would consent to remain and receive training for honest employment.

The standing bugbear of the eighteenth century was the supposed decrease of the population. Montesquieu, writing about the middle of the century, did not hesitate to deduce from a review of the past, and the statistics at his command, the melancholy conclusion that mankind was slowly dying out, and could even point to the period when the race of civilised beings would be extinct. In England the ever unsatisfied demand for soldiers and seamen to prosecute foreign wars led to the gloomiest prognostications of our national downfall. Facts and figures were not at that period readily accessible, and the alarmists had it all their own way. Yet it is clear that the population of England was all this time steadily increasing at the rate, as the century advanced, of as much as 400,000 a year. In London an apparent decrease was at one time revealed by the bills of mortality, but it was evident to all observers that the inhabitants were rapidly increasing in every quarter of the town, and this discrepancy is accounted for by the fact that the ceremony of baptism was very commonly omitted, and that while only church burials were reckoned, a large number of burial-grounds had been leased by Dissenters, in which unregistered accommodation was offered at half-price. In view of these alarms, every encouragement was given to early marriages, premiums were bestowed on large families, and the most impecunious classes were induced to believe that to bring into the world children whom they were unable to support tended to national prosperity. The results of this curious doctrine are even now being felt. Its immediate outcome, induced by frequent infanticide and the heavy infant mortality, was the establishment of the group of lying-in hospitals which date from this period. The justification for the establishment of this class of special hospitals was the grave danger incurred by treating midwifery cases in a general hospital, in immediate proximity to the infection of the other wards. It was never imagined for a moment that any evil would arise from massing together in a separate building women in this condition. The extraordinary zeal shown in promoting these schemes, and the amount of money lavished on them by the public is fully explained by the temper of the times, thus defined by a benevolent writer in advocating the claims of a lying-in charity: "The good of the commonwealth puts in a powerful claim, that this and similar institutions should be countenanced and supported. Nothing is so likely to promote industry and population as the encouragement of matrimony, and nothing will be more likely to encourage it than for the lower orders of the people to see that the rich and great are ready and willing to contribute towards their happiness and support in so honourable a state." The first institutions were, it may be observed, rigidly reserved for married women.

The British Lying-In Hospital, opened by the Duke of Portland in 1749, was the first of its class in London. The advancement of medical science, as well as the relief of distress, formed part of its original plan; and a midwifery school, which cannot fail to have been of the greatest benefit, was attached to it from the beginning. This provided for midwives three or four months' training in the hospital, and instruction not merely in the practice of their art, but also by means of lectures well illustrated with a "large apparatus of preparations," in its theory. An additional feature of this institution was the careful preservation of statistics relating to the death-rate among mothers and infants in the hospital, and the publication of the several results; this course the authorities wisely persevered in, notwithstanding what some persons deemed the "considerable risk lest their publication should at any time be discouraging." There are many indications that the management was exceptionally wide-minded and efficient.

The City of London Lying-in Hospital was founded in the following year, and located at first in apartments rented in Aldersgate and converted into wards. In 1751 it was removed to a better house in the neighbourhood, where it met with such continued success and support that in 1773 a new building was erected for it in the City Road, at that time in an airy country situation. Although the rules of this and the preceding institution provided for the reception of married women only, the claims of the wives of soldiers and seamen serving abroad being specially considered, it appears that frauds were constantly practised by persons of a different class, who procured admission under false pretences. It would seem also that places where absence of regulations tended distinctly to immorality, had been founded, and had even dared to appeal for charitable funds. Accordingly, in the year 1773, an Act was passed, setting forth that some inconvenience having been found to arise from the number of bastard children born in such institutions, all hospitals of this nature were required to obtain a licence from two justices, and to affix in the front of the building "Licensed for the public reception of pregnant women, pursuant to an Act of Parliament passed in the thirteenth year of the reign of King George the Third."

The Queen's Lying-In Hospital, now known as Queen Charlotte's, was founded in 1752 "near the turnpike, entering upon the Uxbridge Road," and did not receive its name until on its removal to Bayswater, about forty years later, the Queen lent it her patronage. A later part of the plan was to afford relief under special regulations to unmarried women also. The affairs of the hospital underwent a good deal of fluctuation, and owing to its terribly high rate of mortality it fell at one time into disuse.

The Westminster Lying-In Hospital, now known as the General Lying-In, was instituted in 1765. It was specially intended for the "wives of poor industrious tradesmen or distressed housekeepers" or of indigent soldiers, of which latter class there were very many in the neighbourhood. Unmarried women were received, but not a second time. Female pupils were received as well as male students, and it is interesting to learn that they paid at the rate of 10s. 6d. a week, exclusive of tea, wine, or porter. An important development of the Lying-in Hospital is indicated in the provision made by this institution for home attendance on those women who could not conveniently be moved or did not wish to leave their families. This will be more fully dealt with in the following article.

It will be seen that the distribution of the four great lying-in hospitals was fairly satisfactory as regards the population. The British Lying-in ministered to a great central and intensely poor district. The City of London, just outside and on the north of the City, was within easy reach of the eastern district. Westminster had its own lying-in charity, and Queen Charlotte's provided for the west.

It is interesting to observe that the generation which so liberally founded and endowed these hospitals proved capable of grappling boldly with the question of unauthorised and mischievous institutions, and that the first school for providing regular instruction from medical men to women engaged in attendance on the sick dates from this period.

It was many years before the dangers attending this class of special hospitals were fully realised, but no new lying-in hospital has been founded in this century.

